# Effects of a Shift of the Signal Peptide Cleavage Site in Signal Peptide Variant on the Synthesis and Secretion of SARS-CoV-2 Spike Protein

**DOI:** 10.3390/molecules27196688

**Published:** 2022-10-08

**Authors:** Zhikai Zhang, Xuan Wan, Xinyue Li, Chengsong Wan

**Affiliations:** 1 BSL-3 Laboratory (Guangdong), Guangdong Provincial Key Laboratory of Tropical Disease Research, School of Public Health, Southern Medical University, Guangzhou 510515, China; 2 Chronic Airways Diseases Laboratory, Department of Respiratory and Critical Care Medicine, Nanfang Hospital, Southern Medical University, Guangzhou 510515, China

**Keywords:** signal peptide, RBD protein, secretion, mutant, SARS-CoV-2

## Abstract

The COVID-19 pandemic is caused by SARS-CoV-2; the spike protein is a key structural protein that mediates infection of the host by SARS-CoV-2. In this study, we aimed to evaluate the effects of signal peptide on the secretion and release of SARS-CoV-2 spike protein. Therefore, we constructed a signal peptide deletion mutant and three signal peptide site-directed mutants. The (H) region and (C) region in the signal peptide of L5F-S13I mutant have changed significantly, compared with wild type, L5F and S13I. We demonstrated the effects of signal peptide on the secretion and synthesis of RBD protein, finding that mutation of S13 to I13 on the signal peptide is more conducive to the secretion of RBD protein, which was mainly due to the shift of the signal peptide cleavage site in the mutant S13I. Here, we not only investigated the structure of the N-terminal signal peptide of the SARS-CoV-2 spike protein but also considered possible secretory pathways. We suggest that the development of drugs that target the signal peptide of the SARS-CoV-2 spike protein may have potential to treat COVID-19 in the future.

## 1. Introduction

At the beginning of 2020, a serious outbreak of pneumonia was attributed to a novel coronavirus [1,2]. Six human coronaviruses (HCoVs), HCoV-229E, HCoV-OC43, HCoV-NL63, HCoV-HKU1, SARS-CoV and MERS-CoV, were known to infect humans [3]. The first four viruses usually cause relatively mild cold-like symptoms in those with a healthy immune system, whereas the latter two viruses are zoonotic viruses and can cause serious respiratory disease and death. The beta-coronavirus SARS-CoV-2 has now become the seventh discrete coronavirus species that is capable of causing human disease [4]. SARS-CoV-2 is easily transmitted and highly pathogenic [5] and COVID-19 was declared a pandemic by the WHO (https://www.who.int/, accessed on 27 July 2022).

The total ssRNA genome of a SARS-CoV-2 strain, isolated from a patient with novel coronavirus pneumonia, was found to contain 29,903 base pairs (GenBank: MN908947.3). Coronavirus spike proteins are involved in binding and fusion of the virus with the host cell membrane, and initial studies showed that the spike protein of SARS-CoV-2 is very similar to that of SARS-CoV. This suggested that angiotensin converting enzyme 2 (ACE2), which was known to be a receptor for SARS-CoV spike protein, may also be an important receptor for the SARS-CoV-2 spike protein [6,7]. Further studies showed that the SARS-CoV-2 spike protein has a higher binding affinity than SARS-CoV spike protein for ACE2, indicating that SARS-CoV-2 may be more invasive than SARS-CoV [8]. The SARS-CoV-2 spike protein has two main domains, S1, which is responsible for binding to the receptor, and S2, which is responsible for fusion with the host cell membrane [9].

A trend towards variation has been observed in SARS-CoV-2. Multiple SARS-CoV-2 variants have been reported, such as the RBD mutation that appears in lineages B.1.1.7 (Alpha), B.1.351 (Beta), P.1 (Gamma), B.1.429 (Epsilon), B.1.617.1 (Kappa), B.1.351 (Beta), P.2 (Zeta), B.1.526 (Iota), B.1.617.2 (Delta) and B.1.1.529 (Omicron) [10,11,12,13]. These studies indicate that genetic variations in the virus lead to changes in the interactions between virus and host and will affect the selection of drugs and treatment regimens in clinical practice.

Many studies have shown that the spike protein is key to viral infection and could potentially be a therapeutic target. Research on the SARS-CoV-2 spike protein, mainly focused on receptor identification [14,15], structural analysis [16], regulation of spike protein binding to its receptor [17,18] and natural products as inhibitors to affect the function of SARS-CoV-2 spike protein [19]. In particular, the receptor binding site of the spike protein had become an important target for the development of SARS-CoV-2 therapeutic antibodies and vaccine design [14,20]. However, there are few reports describing regulation of spike protein expression or processing modifications and release of the protein. In eukaryotic cells, translated proteins must undergo a series of processing modifications and be secreted to become functional proteins with biological activity, and these steps are typically governed by an N-terminal signal peptide [21]. In this study, we investigated the potential effects of signal peptides on the expression and secretion of SARS-CoV-2 spike protein to find novel drug targets for the treatment of COVID-19.

## 2. Results

### 2.1. Bioinformatics Analysis Signal Peptide of SARS-CoV-2 Spike(S) Protein

**Figure 1:**
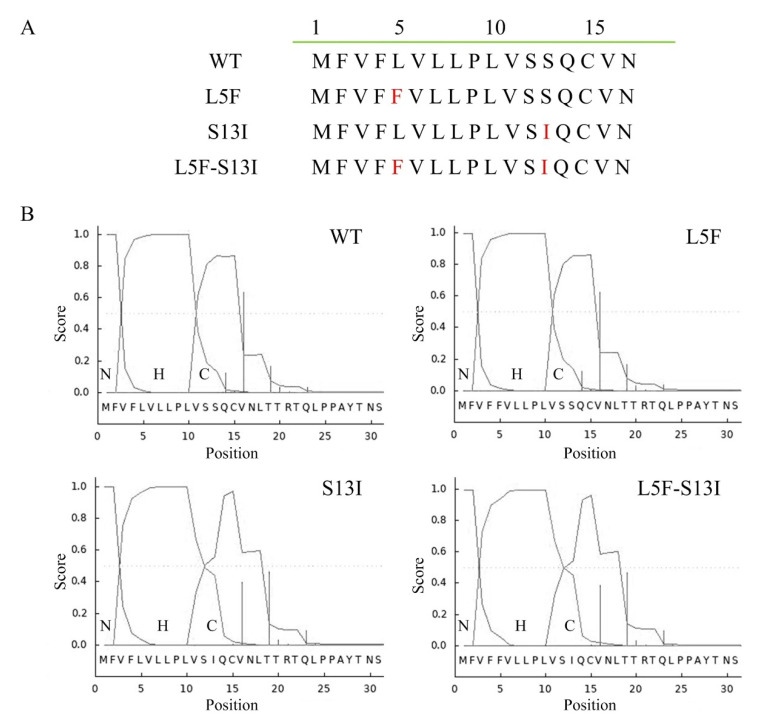
Signal peptide comparison of SARS-CoV-2 spike protein. (**A**) Signal peptide sequences of SARS-CoV-2 spike protein. (**B**) Signal peptide structural analysis by SignalP 3.0. WT means wild-type signal peptide of S protein; The mutants L5F and S13I were present in Iota (B.1.526) and Epsilon (B.1.429) variants; L5F-S13I was a newly constructed double mutant.

Sequences analysis of the signal peptide of SARS-CoV-2 spike protein was shown (Figure 1A). SignalP (Department of Health Technology, Technical University of Denmark, Kgs Lyngby, Denmark) is a freely available web-based tool that uses a deep neural network-based approach to predict the presence and cleavage sites of signal peptides in amino acid sequences from different organisms [22]. The signal peptide structure of SARS-CoV-2 spike protein was analysis using bioinformatics online analysis tools SignalP version 3.0 (http://www.cbs.dtu.dk/services/SignalP-3.0/, accessed on 18 May 2022). The signal peptide of SARS-CoV-2 spike protein can be divided into three regions, an N-terminal (N) region, a central hydrophobic (H) region and a C-terminal (C) region (Figure 1B). The positive charge on the N region of the signal peptide has been shown to contribute to efficient post-translational translocation of small prerequisite proteins [23]. The H region of the signal peptide sequence is mainly responsible for recognition and binding by the signal recognition particle [24]. Comparing with the structures of wild type signal peptide, we found no significant change in the single-point mutant L5F, but significant changes in the (C) region of single point mutant S13I (Figure 1B). It is interesting that the (H) region and (C) region of L5F-S13I mutant have changed significantly, compared with wild type, L5F and S13I (Figure 1B). In addition, after the mutation of S13 to I13, the cleavage position at Q14 and V16 of the region (C) of mutants S13I and L5F-S13I also shifted compared with wild-type and mutant L5F, respectively (Figure 1B) The results indicated that the mutation of signal peptide may affect the expression, secretion and modification of SARS-CoV-2 spike protein.

### 2.2. Signal Peptide Targets SARS-CoV-2 RBD Protein to the Endoplasmic Reticulum

**Figure 2:**
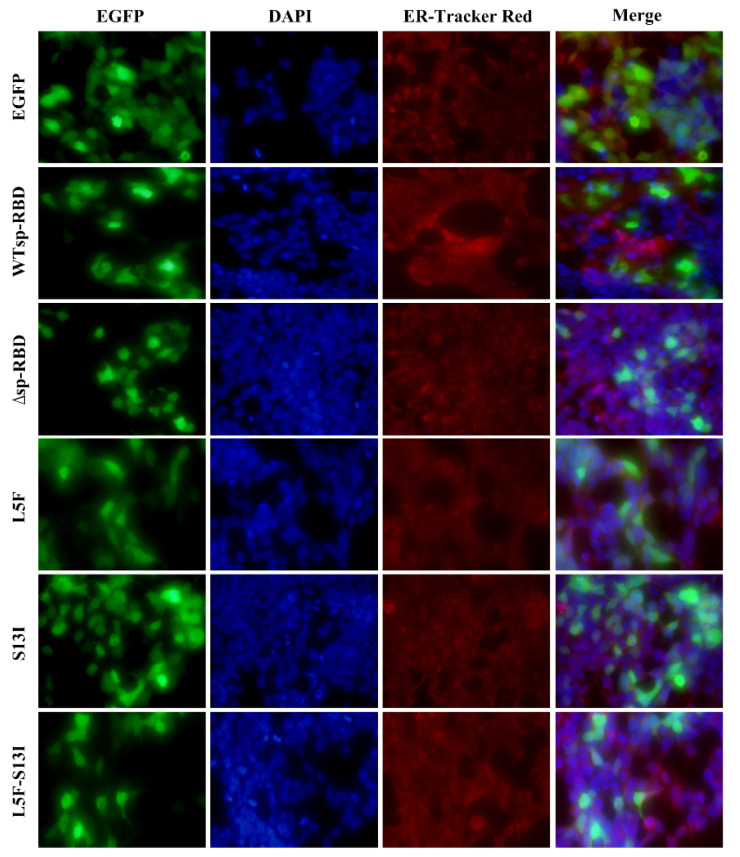
Signal peptides mediate the localization of SARS-CoV-2 RBD protein. WT means wild-type signal peptide of S protein; The mutants L5F and S13I were present in Iota (B.1.526) and Epsilon (B.1.429) variants; L5F-S13I was a newly constructed double mutant. The fluorescence for EGFP (green), DAPI (blue), ER-Tracker-Red (red) and the merge of the three channels are displayed.

In order to determine the effect of signal peptides on the expression of the RBD protein and the localization of the endoplasmic reticulum, recombinant plasmids pEGFP-ΔRBD, pEGFP-RBD, pEGFP-RBD-L5F, pEGFP-RBD-S13I and pEGFP-RBD-L5F-S13I were transiently transfected into HEK293T cells, respectively, and then cell fixation, DAPI, red fluorescent probe staining of endoplasmic reticulum and fluorescence microscopy were performed. As shown in Figure 2, EGFP protein and all fusion proteins could be expressed normally, indicating that the signal peptide had no significant effect on the expression of RBD protein. However, after the deletion of signal peptides, EGFP protein and ΔRBD-EGFP fusion protein were mainly distributed in the nucleus, and only a small amount is distributed in the cytoplasm. The RBD-EGFP protein and mutant RBD-EGFP fusion protein containing signal peptides are mainly distributed in the cytoplasm, and a small amount is distributed in the nucleus (Figure 2). In addition, there are many more proteins in the cytoplasm of mutations S13I and L5F-S13I, compared to wild type and mutant L5F, which may be mainly due to the mutation at the S13 site in the signal peptide. These results suggest that the (C) region amino acid sites in signal peptide have an important influence on the localization of the endoplasmic reticulum of the SARS-CoV-2 spike protein, which means that the (C) region sites may become important candidate targets for drug design.

### 2.3. Signal Peptide Promotes the Secretion Levels of SARS-CoV-2 RBD Protein

**Figure 3:**
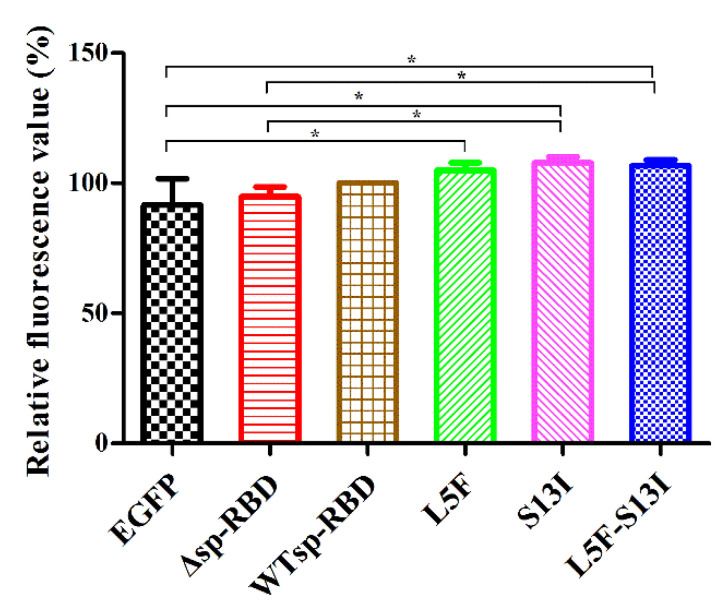
Analysis of RBD protein release regulated by signal peptide. WT means wild-type signal peptide of S protein; the mutants L5F and S13I were present in Iota (B.1.526) and Epsilon (B.1.429) variants; L5F-S13I was a newly constructed double mutant. Statistical significance was defined as *p* < 0.05, and * *p* < 0.05.

To further evaluate the effects of signal peptide on the secretion and release of SARS-CoV-2 spike protein, the green fluorescence intensity in cell culture supernatants were detected to determine the secretion of the fusion protein. After the HEK293T, EGFP, Δsp-RBD, WTsp-RBD, L5F, S13I and L5F-S13I cell lines were normally cultured for 48 h, the culture supernatant was collected by low speed centrifugation, and then the green fluorescence intensity in the supernatant was detected by a multifunctional microplate reader. The results showed that the relative fluorescence intensity in the supernatant of EGFP and Δsp-RBD cells were lower, which was significantly different from that in the supernatant of mutant L5F, S13I and L5F-S13I cells (Figure 3). This implies that EGFP and Δsp-RBD proteins could not be secreted to the extracellular without the signal peptide, while WTsp-RBD, L5F, S13I and L5F-S13I proteins could be secreted extracellular after modification mediated by signal peptide. The relative fluorescence value of mutant S13I and L5F-S13I was significantly, compared to the mutant Δsp-RBD, which was also suggested that mutation of S13 to I13 on the signal peptide is more conducive to the secretion of RBD protein.

## 3. Discussion

Recent progress in biology and medicine has enabled the development of many new drugs for the treatment and prevention of diseases. It is interesting that many membrane-bound and secretory proteins in the conventional secretory pathway have been identified as potential targets for drug design [25]. In the conventional secretory pathway, signal peptides located at the N-terminus of precursor proteins guide these proteins into the ER to complete post-translational processing and modification [21,26]. Some research groups have, therefore, chosen to focus on identifying small molecules that can specifically bind to the signal peptide, and thus inhibit the normal expression of the target protein [25,27,28]. Such studies have clearly demonstrated that signal peptides can be used to design drugs for specific target proteins.

It has been shown that increasing the hydrophobicity of the (H) region of a signal peptide can enhance the production of full length monoclonal antibodies [29]. The effects of a variety of different signal peptides on antibody yield were evaluated in an attempt to increase production of VRC01, a broadly neutralizing antibody against HIV [30]. As another example, the insertion of a cleavable leucine-rich signal peptide into olfactory receptors has been shown to increase expression of the receptors on the surface of HEK293T cells [31]. In our research, the mutants L5F and S13I were present in SARS-CoV-2 of Iota (B.1.526) and Epsilon (B.1.429) variants. However, although the mutant of L5F and L5F-S13I on the signal peptide of RBD protein, the expression of proteins could not significantly increase (Figure 2 and Figure 3). Studies had shown that the positive charge on the (N) region of the signal peptide has been shown to contribute to binding by the signal recognition particle and efficient post-translational translocation of small prerequisite proteins [32,33]. It was suggested the (N) region of the signal peptide plays a key role in protein expression. Because the mutant L5F was in the (N) region of the signal peptide, we hypothesized that the L5 sites of the signal peptide may not have significant effects on the expression of SARS-CoV-2 spike protein.

Protein glycosylation is one of the most important forms of post-translational modification and 50–70% of cellular proteins may be glycosylated [34]. Glycosylation plays a role in regulating the localization, function and activity of proteins in tissues and cells [35,36,37]. In eukaryotes, glycosylation of most cellular proteins takes place along the secretory pathway, which begins in the ER and is completed in the Golgi apparatus [38]. The N-terminus of the SARS-CoV-2 spike protein contains a signal peptide (Figure 1), which indicates that synthesis, processing and release of the spike protein in the ER are mediated by the signal peptide. So far, 22 N-linked glycosylation sites and 17 O-linked glycosylation sites have been identified in the SARS-CoV-2 spike protein [39]. In addition, Veesler et al. though mass spectrometry and structural studies, revealing that the S13I mutation resulted in total loss of neutralization for 10 of 10 NTD-specific mAbs because the NTD antigenic supersite was remodeled by a shift of the signal peptide cleavage site and the formation of a new disulfide bond [40]. Therefore, signal peptide plays an important role in the expression, secretion and modification of SARS-CoV-2 spike protein. In our study, mutants L5F and S13I occur on the signal peptide, and S13I, in particular, could significantly increase the secretory capacity of the RBD protein, which suggests that S13 was a key site on the signal peptide. In addition, the glycosylation of viral structural proteins is closely associated with viral replication, infectivity and the host immune response [41,42,43]. The shielding of receptor binding sites by glycosylation is a common feature of viral glycoproteins and can be observed with SARS-CoV spike protein, HIV-1 envelope protein, influenza virus hemagglutinin and Lassa virus glycoprotein precursor [44]. HIV-1 envelope glycoprotein gp160 is directed to the ER by its signal peptide and the premature cleavage of the mutated gp160, compared with wild-type gp160, results in a virus with significantly reduced adaptability [45].

**Figure 4:**
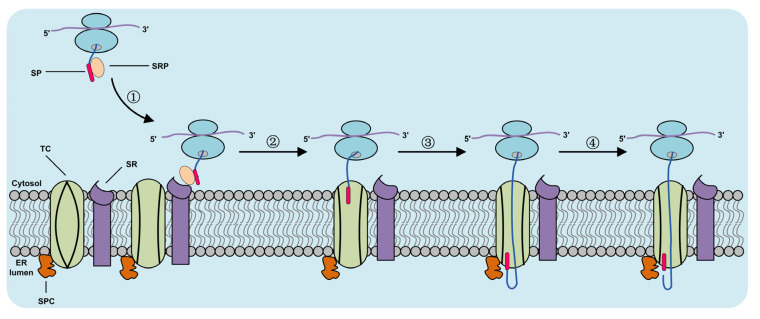
Proposed secretion pathway of SARS-CoV-2 spike protein. ① New polypeptide-SRP-ribosomal complex binding to SR on ER membrane. ② SRP separates from its receptors and promotes the tight binding of ribosomes and ER membranes to protein translocon channel. ③ The extended polypeptide passes through the membrane structure into the ER cavity as translation continues. ④ The signal peptidase inside the ER membrane cavity cleaves the signal peptide after recognizing the signal peptide cleavage site of the polypeptide, and the remaining polypeptides continue to undergo co-translational translocation through the ER membrane. SP: signal peptide. SRP: signal recognition particle. SR: signal receptor. TC: translocon channel. ER: endoplasmic reticulum. SPC: signal peptidase cleavage.

Based on the above analyses, we are convinced that the synthesis and secretion of SARS-CoV-2 spike proteins take place through the conventional secretion pathway (Figure 4). We speculate, therefore, that the signal peptides may play an important role in the synthesis, processing modification and secretion of SARS-CoV-2 spike protein, and thus represent a new target for drug design. Drugs that interfere with the co-translational translocation of new polypeptide chains have the potential to reduce the expression of many cell surface receptors and secretory proteins that are important therapeutic targets [46,47]. For example, the small molecule cyclotriazadisulfonamide (CADA) selectively downregulates the expression of CD4, the primary receptor for human immunodeficiency virus (HIV) and simian immunodeficiency virus, and thus inhibits viral replication and reduces pathogenicity [27,48]. Another small molecule, CAM741, which is an analog of the cycloheptadepsipeptide fungal metabolite HUN-7293, is a signal peptide-selective inhibitor of protein co-translation translocation [28].

## 4. Materials and Methods

### 4.1. Bacterial, Plasmids Construct and Cells Culture Conditions

The *E. coli* DH5α was used to cloning host strain. All bacterial strains in our experiment were cultured in LB medium at 37 °C and kanamycin was added as needed. HEK293T cells were cultured in DMEM (Gibco, Waltham, MA, USA) broth containing 6% fetal bovine serum (FBS) and 1% at 5% CO_2_ and 37 °C. SARS-CoV-2 RBD gene fragment was derived from our previous experiment. The plasmid fragment of pEGFP-N1 was obtained by PCR to construct recombinant expression vector. The signal peptide mutants of SARS-CoV-2 spike protein were constructed by reverse PCR. The primers were listed in Table 1.

### 4.2. RBD Proteins Expressed and Intracellular Fluorescence Assay in HEK293T Cell

The SARS-CoV-2 RBD proteins were expressed by transiently transfecting HEK293T cells with recombinant plasmid pEGFP-ΔRBD (None signal peptide), pEGFP-RBD, pEGFP-RBD-L5F, pEGFP-RBD-S13I and pEGFP-RBD-L5F-S13I. HEK293T cells were placed in a 35 mm confocal dish with 1 × 10^4^ per plate in advance and cultured in 5% CO_2_ cell incubator at 37 °C for 24 h. After the cells grew well, recombinant plasmids were transfected according to the method of PEI transfection reagent. Cell culture medium was discarded after 6 h and a new serum-free medium was used for further culture. After 24 h of culture, the medium were removed and cells were rinsed with PBS buffer twice. Next, the cells were fixed with 4% paraformaldehyde at room temperature for 15 min. Paraformaldehyde was removed and cells were rinsed with PBS 3 times, DAPI dye (Cat. No.: C1005, Beyotime, Shanghai, China) or endoplasmic reticulum red fluorescent probe (Cat. No.: C1041, Beyotime, Shanghai, China) diluted 1000 times were added, and the cells were incubated at room temperature without light for 5 min or 20 min. The cells were rinsed with PBS 3 times, and 1–2 drops of anti-fluorescence quenching agent were added. Photos were taken under the fluorescence microscope after the slides were covered.

### 4.3. RBD Protein Secretion Detection

Recombinant plasmids were transiently transfected HEK293T cells; SMM 293-TI medium (Sino Biological, Beijing, China) was used to replace DMEM medium before transfection. After 48 h culture, cell fragments were removed and supernatant was collected by centrifugation at 12,000× *g* for 3 min at room. The 200 μL supernatant sample was added to a 96-well black plate, and the fluorescence absorption value was read by the microplate instrument. The detection conditional excitation wavelength was 485 mm, and the transmission wavelength was 515 mm. The relative fluorescence value is expressed by the following Equation (1):

(1) $$Rf=\frac{S-N}{P-N}\times 100\%$$

*Rf*: the relative fluorescence value;
N: fluorescence value of negative control (HEK293T);
P: fluorescence value of positive control (pEGFP-RBD);
S: fluorescence value of target sample.

### 4.4. Statistical Analysis

For all experiments, unless stated otherwise, three independent were performed. Statistical analysis were drawn using GraphPad Prism software (version 5.0, GraphPad Software, San Diego, CA, USA). Significant differences were determined by one-way analysis of variance followed by the Tukey’s multiple comparison test. Statistical significance was defined as *p* < 0.05.

## 5. Conclusions

Signal peptides have huge potential as drug design targets and are also very important in vaccine production. This is the first study to report about the signal peptide of SARS-CoV-2 spike proteins. We analyzed the signal peptide structure of SARS-CoV-2 spike protein and found that the mutants L5F and S13I could change regions. In particular, the mutant S13I could shift the signal peptide cleavage site in the (C) region on the signal peptide. Compared with wild type and mutant L5F, the mutants S13I and L5F-S13I can promote the secretion of SARS-CoV-2 RBD protein. In order to further clarify the role of L5F and S13I mutations on signal peptides, it was found that S13 is a key site on signal peptides that may play an important role in the expression, modification and secretion of SARS-CoV-2 spike protein. Moreover, we propose that signal peptides may be effective targets for the design of drugs to treat SARS-CoV-2 infections.

## Figures and Tables

**Table 1:** Primers used in this study.

Primers Name	Sequence (5′–3′)
pEGFP-F	CATCATCACCATCACCATGGATCCACCGGTCGCCACCATGGTG
pEGFP-R	GGTGGCGAATTCGAAGCTTGAGCTC
∆RBD-F	GAGCTCAAGCTTCGAATTCGCCACCATGAATATTACAAACTTGTGCCCTTTTG
∆RBD-R	TGGATCCATGGTGATGGTGATGATGCTCAAGTGTCTGTGGATCACGGAC
RBD-F	CTTGTTTTATTGCCACTAGTCTCTAGTCAGTGTGTTAATATTACAAACTTGTGCCCTTTTG
RBD-R	CTAGAGACTAGTGGCAATAAAACAAGAAAAACAAACATGGTGGCGAATTCGAAGCTTGAGCTC
L5F-F	TTGTTTTTTTTGTTTTATTGCCACTAGTCTCTAGTC
L5F-R	CAATAAAACAAAAAAAACAAACATGGTGGCGAATTCG
S13I-F	CTAGTCTCTATTCAGTGTGTTAATATTACAAACTTGT
S13I-R	ACACACTGAATAGAGACTAGTGGCAATAAAACAAGA

## Data Availability

Not applicable.
